# Characterization of a Novel Bile Alcohol Sulfate Released by Sexually Mature Male Sea Lamprey (*Petromyzon marinus*)

**DOI:** 10.1371/journal.pone.0068157

**Published:** 2013-07-09

**Authors:** Ke Li, Cory O. Brant, Michael J. Siefkes, Hanna G. Kruckman, Weiming Li

**Affiliations:** 1 Department of Fisheries and Wildlife, Michigan State University, East Lansing, Michigan, United States of America; 2 Great Lakes Fishery Commission, Ann Arbor, Michigan, United States of America; INRA-UPMC, France

## Abstract

A sulphate-conjugated bile alcohol, 3,12-diketo-4,6-petromyzonene-24-sulfate (DKPES), was identified using bioassay-guided fractionation from water conditioned with sexually mature male sea lamprey (*Petromyzon marinus)*. The structure and relative stereochemistry of DKPES was established using spectroscopic data. The electro-olfactogram (EOG) response threshold of DKPES was 10^−7^ Molar (M) and that of 3-keto petromyzonol sulfate (3 KPZS; a known component of the male sea lamprey sex pheromone) was 10^−10^ M. Behavioural studies indicated that DKPES can be detected at low concentrations by attracting sexually mature females to nests when combined with 3 KPZS. Nests baited with a mixture of DKPES and 3 KPZS (ratio 1∶29.8) attracted equal numbers of sexually mature females compared to an adjacent nest baited with 3 KPZS alone. When DKPES and 3 KPZS mixtures were applied at ratios of 2∶29.8 and 10∶29.8, the proportion of sexually mature females that entered baited nests increased to 73% and 70%, respectively. None of the sexually mature females released were attracted to nests baited with DKPES alone. These results indicated that DKPES is a component of the sex pheromone released by sexually mature male sea lamprey, and is the second biologically active compound identified from this pheromone. DKPES represents the first example that a minor component of a vertebrate pheromone can be combined with a major component to elicit critical sexual behaviors. DKPES holds considerable promise for increasing the effectiveness of pheromone-baited trapping as a means of sea lamprey control in the Laurentian Great Lakes.

## Introduction

Bile alcohols and bile acids are amphipathic multifunctional end products of cholesterol metabolism in vertebrates [1], [2]. In vertebrate animals, bile alcohols are synthesized in hepatocytes, conjugated by esterification with sulfate to form bile salts, converted to strong acids at the terminal carbon of the side chain, and shown to have substantial structural diversity across species [1]–[3]. Recent studies have shown that the olfactory epithelia of teleost fishes and jawless vertebrates are acutely sensitive to bile acids [4]–[6]. Bile salt molecules are ideal for chemical communication because of their stability and water solubility [7].

The sea lamprey (*Petromyzon marinus*) provides a useful model for studying intraspecific chemical communication mediated by bile salts [6]. These semelparous, jawless vertebrates rely on bile salts released from conspecifics to coordinate reproduction [8]–[11]. Previous research has demonstrated that 3-keto petromyzonol sulfate (3 KPZS), a bile salt derivative, is a potent sex pheromone released by sexually mature male sea lamprey, and elicits upstream movement in sexually mature females [12]–[14].

Behavioral studies have indicated that there are additional components of the male sex pheromone that play roles in inducing additional behaviors, such as attraction and nest retention, in sexually mature females [15]. During in-stream behavioral bioassays, water conditioned with sexually mature males, which presumably contained all the components of the male sea lamprey sex pheromone, attracted more sexually mature females to artificial nests, retained females at nests longer, and induced a greater number of nesting and spawning behaviors than when 3 KPZS alone was applied, indicating that other constituents may be critical for these behaviors [15]. The male sea lamprey sex pheromone is hypothesized to contain additional components, some of which may function more locally around nests to attract female mates [16]. This study reports the structure and biological activity of a novel bile salt, 3,12-diketo-4,6-petromyzonene-24-sulfate (DKPES), recently isolated from the male sea lamprey sex pheromone, which represents the first example of the importance of minor components in vertebrate pheromone communication.

## Results

### Structure Elucidation of DKPES

DKPES was isolated as a colorless oil. The molecular formula, C_24_H_33_NaO_6_S, was deduced from its HRESIMS (*m*/*z* 495.1782 [M+Na]+), (See Supplementary Data), which corresponds to C_24_H_33_Na_2_O_6_S (calcd, 495.1793). The MS/MS fragmentation showed a 96.9603 fragment, which corresponds to HSO_4_^−^. The ^13^C chemical shift for C-24 (*δ*_C_ 69.4 ppm) was similar to a sulfate half-ester OSO_3_Na [17]. The ^1^H NMR spectrum, (See Figure S1, S2, S3, S4, S5, S6, S7), showed two deshielded methyl singlets (*δ*_H_ 1.17, s, H-18; *δ*_H_ 1.24, s, H-19) and one methyl doublet (*δ*_H_ 0.87, d) with chemical shifts corresponding to methyl groups H_3_-21. The results of the ^1^H NMR spectrum revealed the sulfated steroidal nature of DKPES. The ^13^C NMR spectrum of DKPES displayed 24 carbon signals. Two carbonyl carbon signals appeared at *δ*_C_ 201.9 (qC) and 215.5 (qC), and four olefin carbon resonances were located at *δ*_C_ 124.5 (CH), 165.7 (qC), 129.4 (CH), and 141.4 (CH). Using the gHSQC spectrum (See Supplementary Data), the remaining carbon resonances were three methyl carbons, eight methylene carbons, five methine carbons, and two quaternary carbons, indicating that DKPES was a tetracyclic structure with eight degrees of unsaturation.

**Figure 1:**
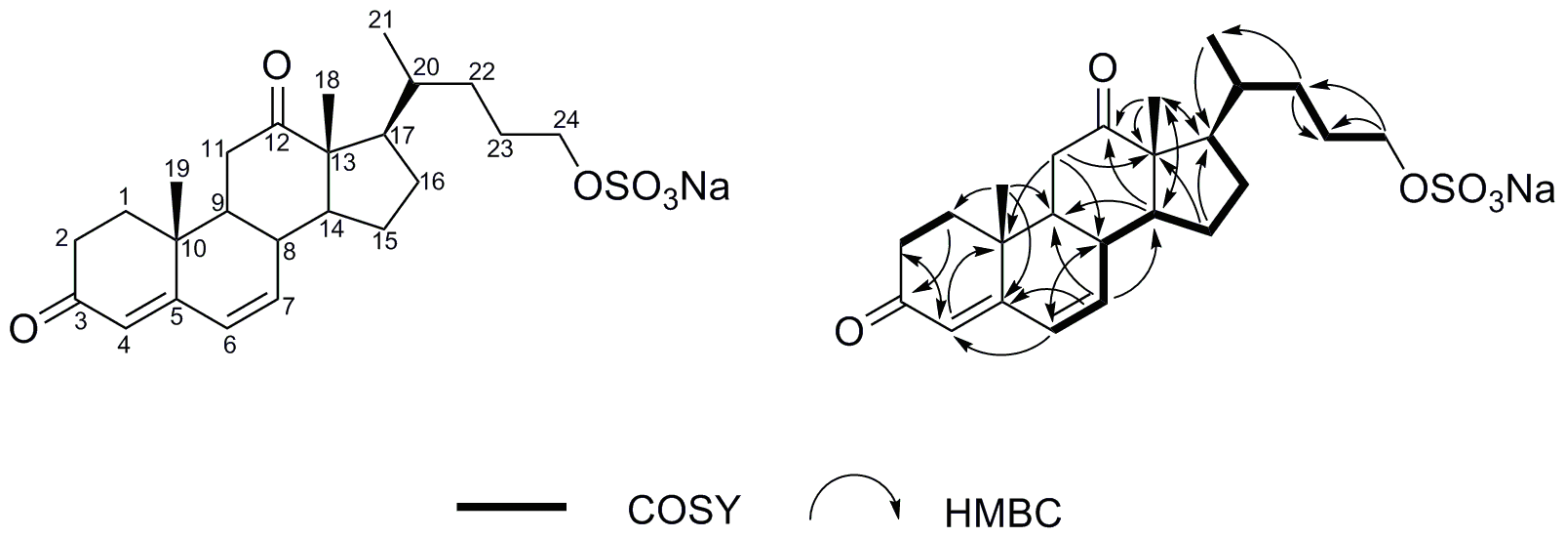
Selected key correlations of compound DKPES.

The major part of the tetracyclic steroidal backbone could be assembled through the interpretation of COSY and HMBC correlations. The gCOSY experiment showed two spinning systems (indicated in bold in Figure 1), while a series of gHMBC correlations displayed the connection of these fragments (Figure 1). Characteristic gHMBC correlations, exhibited by the two methyl singlets Me-18 and Me-19, confirmed the cyclization of four rings and the location of a ketone at position of C-12 (Figure 1).The correlations from H_2_-2 to C-4, from H-4 to C-2, C-4, and C-6, from H-6 to C-4 and C-8, and from H-7 to C-5 and C-9 indicated that the conjugated ketene was placed at C-4, Δ^4(5)^, and Δ^6(7)^ (Figure 1). The carbon ring skeleton of DKPES was originally known to be one of the minor unsaturated acidic metabolites degraded by cholic acid under anaerobic conditions by *Pseudomonas* sp [18]. An overall comparison of ^1^H and ^13^C NMR data (Table 1) revealed that the main tetracyclic skeleton of DKPES was similar to 21-hydroxypregna-4, 6-diene-3, 12, 20-trione and 20*R*-hydroxypregna-4, 6-diene-3,12-dione, which had been isolated from the bark and twigs of *Nerium oleander* L. from the family of Apocyaceae [19]. The Compound DKPES possessed the same conjugated ketene system in the ring A and B, but this ring skeleton with a C-5 side chain had not been discovered until now. The characterization of the DKPES side chain was based mainly on the interpretation of its 2D NMR and by comparing corresponding parts to the model compound 3 KPZS, previously isolated from sexually mature male sea lamprey [8]. The linkage of the ring skeleton and side chain was based on the H-20/H-17 COSY correlation, as well as the cross peaks from H-21 to C-17 in the HMBC spectrum. The planar structure of DKPES was determined to be 3,12-diketo-4,6-choladiene-24-sulfate.

**Table 1:** NMR Spectroscopic data (600 MHz, CD_3_OD) for DKPES.

no.	*δ*_H_ (mult., *J* in Hz)	*δ*_C_
1	1.59 (m);2.63 (ddd, 5.5, 14.4, 18.1)	34.6 (CH_2_)
2	1.95 (m)2.39 (m)	34.7 (CH_2_)
3		201.9 (qC)
4	5.70 (s)	124.5 (CH)
5		165.7 (qC)
6	6.26 (dd, 2.3, 10.8)	129.4 (CH)
7	6.22 (dd, 2.4, 10.2)	141.4 (CH)
8	2.76 (t, 10.8)	38.5 (CH)
9	1.58 (m)	54.4 (CH)
10		37.9 (qC)
11	2.15 (dd, 4.4, 12.3);2.82 (dd, 13.6, 12.4)	38.9 (CH_2_)
12		215.5 (qC)
13		59.3 (qC)
14	1.50 (m)	57.1 (CH)
15	1.95 (ddd, 13.4, 5.2, 1.9)	24.7 (CH_2_)
16	2.02 (m)	28.5 (CH_2_)
17	2.01 (m)	48.1 (CH)
18	1.17 (s)	11.8 (CH_3_)
19	1.24 (s)	16.2 (CH_3_)
20	1.38 (m)	36.9 (CH)
21	0.87 (d, 7.2)	19.5 (CH_3_)
22	1.58 (m)	32.7 (CH_2_)
23	1.61 (m); 1.77 (m)	27.4 (CH_2_)
24	3.97 (t, 6.4)	69.4 (CH_2_)

**Figure 2:**
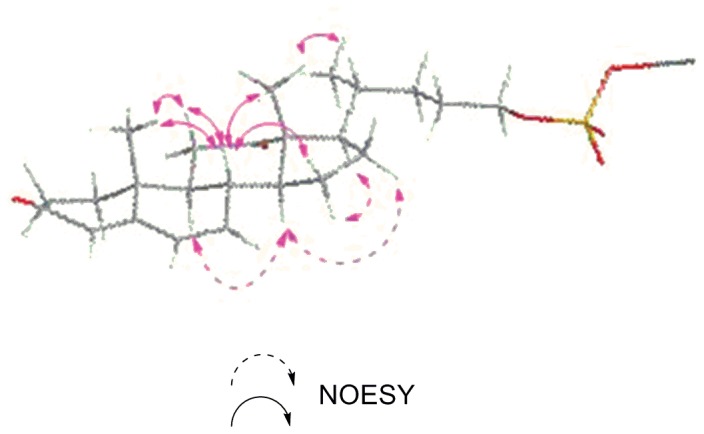
Stereostructure and key NOESY correlations of DKPES.

The relative configuration of the DKPES tetracyclic steroidal skeleton was considered to possess an all-*trans* arrangement, typical of cholesterols, and was supported by coupling constant values and NOESY analysis (Figure 2). The coupling constant between H-8 and H-9 (*J* = 10.8 Hz) implied a *trans* configuration at the junction between B and C rings. The NOESY correlation observed from H-18 to H-8, H-11*β*, H-21, from H-14 to H-9, and H-16 indicated the relative configuration for each ring junction to be *trans* [20]. The 17*β-*orientation and 20R configuration of the side chain was assigned by NOESY correlation of the H_3_-18 methyl group with H-20. The chemical shifts and multiplicities of C-17/H-17, C-18/H-18, C-20/H-20, and C-21/H_3_-21 in the ^13^C NMR and ^1^H NMR spectra were very similar to those of many metabolites isolated from several higher fungi [21]. Using the evidence listed above, the configuration at C-20 was established as R. The relative configuration of this compound has been determined (Figure 2).

### Electro-olfactogram (EOG) Responsiveness

**Figure 3:**
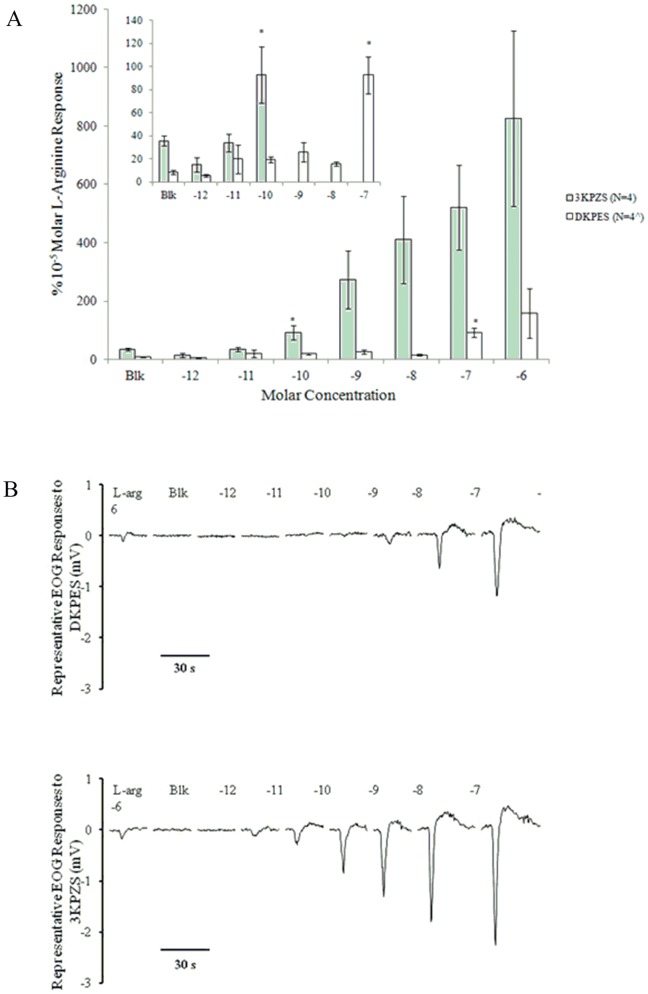
Electro-olfactogram (EOG) responses to lamprey bile salts. A. Semi-logarithmic plots of EOG responses. Semi-logarithmic plots of EOG response in millivolts (mV) to a 10^−5^ Molar _L_-arginine standard (_L_-arg), blank water control (Blk), and increasing concentrations of DKPES and 3 KPZS are presented in the top graph. Semi-logarithmic plots of EOG concentration-responses to DKPES and 3 KPZS as a percentage of the _L_-arg standard are presented in the bottom graph. The response thresholds for DKPES and 3 KPZS are the lowest concentrations that elicit a response significantly greater than the blank water controls (Blk) and are depicted by *asterisks* (Student’s *t*-test, P<0.05). The inset shows a detailed view of the response thresholds. Numbers by the abbreviation indicate sample sizes. The *carat* indicates that the sample size was not achieved for all odor concentrations (see Table 2). *Vertical bars* represent one standard error. B. Representative EOG responses of an adult sea lamprey to 3 kPZS and DKPES in concentrations between 10^−12^ and 10^−8^ molar. The olfactory epithelium was exposed to each odorant for 5 sec. L-arg: L-arginine at 10^−5^ molar. Blk: blank water. The number above each EOG trace is the logarithmic value of the molar concentration of bile salts applied to the sensory epithelium.

The olfactory epithelia of adult female sea lamprey were stimulated by DKPES. The mean response to the _L_-arginine standard was 0.367+/−0.070 mV (mean +/− standard error, SE) during DKPES testing and 0.401+/−0.176 mV during 3 KPZS testing. The mean response to the blank water control was 0.048+/−0.014 mV during DKPES testing and 0.108+/−0.032 mV during 3 KPZS testing. Responses (in mV) to the L-arginine standard, blank water control, and increasing concentrations of DKPES and 3 KPZS are presented in Table 2 and plotted in Figure 3. Concentration-response relationships of the positive control, 3 KPZS, and the test compound, DKPES, were plotted as percentages of the _L_-arginine standard (Figure 3). All responses to 3 KPZS and DKPES ranged from 0% to 1,705%. The response threshold for DKPES (N = 4) was 10^−7^ Molar (M; 92.7% +/−15.9%, mean +/− SE; Student’s t test, P<0.01; Figure 3) and the threshold for 3 KPZS (N = 4) was 10^−10^ M (92.8% +/−24.2%; P<0.04). All DKPES and 3 kPZS responses compared to the blank water controls are presented in Table 2. The concentration-response curve for DKPES was much shallower and did not show a steep increase when compared to the curve generated using 3 KPZS (Figure 3).

**Table 2:** Electro-olfactogram responses to a 10^−5^ Molar (M) _L_-arginine standard (_L_-arg), blank water control (Blk) and increasing concentrations of DKPES and 3 KPZS as raw responses in millivolts (mV) and percentages of a 10^−5^ M _L_-arginine standard.

	DKPES						3 KPZS					
Treatment	*N*	MeanResponse (mV)	SE (mV)	MeanResponse (%)	SE (%)	*P*	*N*	Mean Response (mV)	SE (mV)	MeanResponse (%)	SE (%)	*P*
L-arg	5	0.367	0.070	–	–	–	4	0.401	0.176	–	–	–
Blk	4	0.048	0.014	8.7	1.9	–	4	0.108	0.032	35.5	4.4	–
10^−12^ M	2	0.017	0.010	5.5	1.2	0.31	4	0.092	0.073	15.0	6.5	0.08
10^−11^ M	2	0.095	0.071	20.0	12.5	0.22	4	0.159	0.081	33.8	7.7	0.44
10^−10^ M	3	0.056	0.023	19.2	2.5	0.04	4	0.320	0.100	92.8	24.2	0.04
10^−9^ M	4	0.110	0.025	26.0	8.3	0.08	4	0.782	0.220	273.3	99.0	0.04
10^−8^ M	3	0.174	0.094	15.8	1.8	0.10	4	1.226	0.347	411.0	149.4	0.04
10^−7^ M	4	0.614	0.201	92.7	15.9	0.01	4	1.657	0.426	519.8	145.0	0.02
10^−6^ M	2	0.912	0.332	158.1	85.0	0.17	4	2.519	0.726	826.0	300.9	0.04

*N* is the sample size for each treatment. SE is the standard error. *P*-values were generated using a Student’s *t*-test.

### Behavioral Responses to DKPES

In 2010, mixtures of DKPES:3 KPZS had an influence on both upstream movement (logistic regression; *X* ^2^_3_ = 15.83, *P = *0.001), and nest selection (*X* ^2^_3_ = 10.13, *P = *0.017). Nests baited with DKPES and 3 KPZS at a ratio of 1∶29.8 attracted 43% of females, which was not different from the distribution seen when 3 KPZS alone was applied to both nests during control trials (two-tailed; *t* = 0.39, *P* = 0.699). When the DKPES and 3 KPZS mixture was applied at ratios of 2∶29.8 and 10∶29.8, the proportion of females that entered the ratio-baited nest increased to 73% (*t* = 2.23. *P = *0.026) and 70% (*t* = 1.96, *P = *0.050), respectively (Table 3). While upstream movement differed across mixture treatments, compared to 3 KPZS controls, the difference is likely due to the time frame at which each mixture was tested (see methods).

**Table 3:** Preference responses of sexually mature female sea lamprey to mixtures of DKPES:3 KPZS administered to stream nests.

Treatment	Trials	Released (*N*)	Upstream	Treatmentnest
Control	12	125	47% A	44% A
DKPES:3 kPZS (1∶29.8)	11	107	33% B	43% A
DKPES:3 kPZS (2∶29.8)	4	38	58% A	73% B
DKPES:3 kPZS (10∶29.8)	8	75	27% B	70% B
		X^2^	15.83	10.13
		df	3	3
		*P*-value	0.001	0.018

Treatments include: Control (synthesized 3 KPZS 5×10^−13^ M applied to both nests), and three separate ratios (*v*:*v*) of 3,12-diketo-4,6-petromyzonene-24-sulfate (DKPES) and 3-keto petromyzonol sulfate (3 KPZS) applied to one nest (Treatment nest), while an equal concentration of 3 KPZS alone (5×10^−13^ M) was applied to the adjacent nest. Treatment nests and control nests were alternated per trial. *N* refers to the total number released for each treatment. *Upstream* refers to the percentage of released animals that moved upstream and entered a nest. *Treatment nest* refers to the percentage of animals that moved upstream and then entered the nest activated with each treatment. Distribution data were evaluated with logistic regression. Treatments that share a letter are not significantly different (two-tailed *t* test; α = 0.05).

In 2011, when DKPES was tested alone in the stream, none of the released animals (*N* = 61) moved upstream to the nests. When the DKPZS and 3 KPZS mixture was applied at a ratio of 10∶29.8 to one nest and sexually mature male washings (SMW) to the adjacent nest, of the 56 females released, two entered the ratio-baited nest and 12 entered the SMW baited nest (data not shown). These results consisting of inadequate participants in 2011 were excluded from Table 3. Since 3 KPZS has previously been shown to be the main compound that stimulates upstream movement in sexually mature females [15], we did not expect upstream movement without 3 KPZS in the system. Stream temperatures in the upper Ocqueoc River showed little variation throughout both years of field experiments (*X ± SE* = 21.6±0.5°C). Stream discharge averaged 0.48±0.08 cms.

## Discussion

Bile salts are the major end metabolites of cholesterol, which are produced by every class of vertebrate animal and show remarkable structural diversity across species [1]–[3]. Although jawless vertebrate animals have predominantly C_27_ bile alcohols [1], C_24_ bile alcohols such as PZS and 3 KPZS, a type of bile salt not found in any other animal species to date, have been isolated from sea lamprey. DKPES is a new addition to the sea lamprey bile salt family. Its structure characteristic, a steroid nucleus bearing a 3,12-diketo-4,6-diene, is quite rare in known natural products. The two basic structural components of bile salts are the 19-carbon (C_19_) steroid nucleus and the side chain. In all bile salts characterized to date, the cyclopentanophenanthrene nucleus is fully saturated [22]. All bile salts have hydroxyl groups at C-3 and C-7. Additional common sites of hydroxylation are at C-12 and C-16, but other sites of hydroxylation have not been found. DKPES may have undergone dehydrogenation at C-3, C-7, and C-12 to an oxo group. To the best of our knowledge, the 3, 12-diketo-4, 6-diene-steroid nucleus and the unconventional 24-sulfate side chain is unique to DKPES and is the first sulfated bile alcohol known to possess a conjugated ketene in the steroidal ring at position 3, 4, and 6.

Electrophysiological and behavioral results of this study indicate that DKPES is a component of the sex pheromone released into water by mature male sea lamprey. Male responses to DKPES were not tested given that males did not respond behaviorally to SMW (containing DKPES) [12] or 3 kPZS [8] in previous experiments. The female sea lamprey olfactory epithelium detects DKPES, with an EOG detection threshold of 10^–7 ^M. The response threshold of DKPES during behavioral experiments was lower than in EOG recordings. This is common when EOG and behavioral experiments are combined in research for the following reasons: 1) test subjects are anesthetized during EOG, making conditions less comparable to behavioral trials, 2) pheromone mixtures can produce more dramatic behavioral responses compared to single components (we did not test mixtures during EOG experiments), and 3) sensitivity to odorants in fishes varies across different conditions – *i.e.* stream water, natural light cycle, natural stream velocity, compared to filtered well water for EOG [10], [23], [24]. More importantly, mixtures of DKPES and 3 KPZS at a ratio of either 2∶29.8 or 10∶29.8 were more potent than 3 KPZS alone in attracting and retaining sexually mature females in artificial nests. These results are similar to many known cases in insect pheromone communication where a mixture of compounds that compose the entire pheromone must be present to induce strong preference responses from conspecifics [25], [26].

The mixture of DKPES and 3 KPZS at a ratio of 1∶29.8 was not more attractive than 3 KPZS alone, and DKPES by itself did not show observable effects in behavioral test settings. Whether this reflects yet another well known principle of insect pheromone communication, that the pheromone components must be presented in the exact ratio as in natural pheromones to be behaviorally effective, warrants further examination. Future studies should focus on the accurate estimate of the native ratio of DKPES and 3 KPZS, and on systematically testing their mixture at a range of ratios. Another interesting pursuit would be to search for additional pheromone components from male sea lamprey, as results indicate that washings collected from sexually mature males are much more effective in retaining females than the active mixture of DKPES and 3 KPZS tested, clearly indicating that the washings contain either unknown active components, or contain DKPES and 3 KPZS at a more effective ratio than those tested in this study.

Previous research has demonstrated that blends of chemicals are often employed as pheromones in many species of insects and vertebrate animals [25]–[27]. DKPES is the first example of a minor component of a vertebrate pheromone to show critical function in pheromone communication.

## Materials and Methods

### Animals

All sea lamprey research was conducted in conformity with the Public Health Service (PHS) Policy on Humane Care and Use of Laboratory Animals, incorporated in the Institute for Laboratory Animal Research Guide for Care and Use of Laboratory Animals, as well as in accordance with animal use form #05-09-088-00, approved by the Michigan State University Institutional Animal Use and Care Committee. Adult sea lamprey were trapped in tributaries of the Laurentian Great Lakes during spawning migrations, transported to the US Geological Survey Hammond Bay Biological Station, Millersburg, Michigan, USA, and held in flow through raceways that were 1.83 m wide by 0.61 m deep by 15.24 m long. To produce sexually mature males for pheromone extraction, or sexually mature females for field tests, sexually immature adult sea lamprey were moved to acclimation cages constructed of polyurethane mesh and polyvinyl chloride pipe (0.5 m^3^) located in the lower Ocqueoc River, Millersburg, Michigan, USA. Sea lamprey were monitored daily for sexual maturation. Spermiation of males was determined by checking their seminal fluid, and ovulation of females was determined by applying light pressure to the lower abdomen of each female and observing the free-flowing release of oocytes from the urogenital pore [28].

Pre-migratory adult female sea lamprey for Electro-olfactogram (EOG) recording were collected in December and January by commercial fishers in Lake Huron and transported to Michigan State University, East Lansing, Michigan, USA. Females were held in flow-through tanks (254 L) supplied with well water chilled to ∼ 6°C. EOG recordings were conducted in January 2010.

### Extraction of Sea Lamprey Conditioned Water

Sexually mature male sea lamprey were held in a 0.25 m^3^ Bonar tank (Promens Saint John Inc., New Brunswick, Canada) supplied with ∼ 50 L of aerated Lake Huron water maintained at 16–18°C. Throughout each night from 14 June to 30 July, 2009, the tank flow was turned off so that male conditioned water could be collected. Between15–30 sexually mature males were placed in the tank at a time to reduce crowding and prevent oxygen depletion. Each morning males were removed (and placed into an adjacent tank with flowing water) and the conditioned water was used for solid phase extraction. The conditioned water was passed through a bed of 2 kg of Amberlite XAD 7 HP resin, contained in a series of four 2.5 L-capacity glass columns (Ace Glass Inc., Vineland, New Jersey, USA). Load speeds were maintained between 400 and 600 mL×min^–1^. Pheromone components were eluted with 10 L of methanol, followed immediately by 5 L of acetone. The solvent was removed under reduced pressure at <40°C by roto-evaporation (Buchi Rotovapor®, Flawil, Switzerland). Aqueous solvent was stored at −80°C. For fractionation, extract (4.2 L) was thawed and concentrated by lyophilization (Labconco Corporation, Kansas, USA). The residue was suspended in methanol and filtered. Filtrates were collected and concentrated under reduced pressure at <40°C by roto-evaporatoration. Roto-evaporated filtrates produced 3.1 g of dark residue.

### Isolation of DKPES

To show the feasibility of using an activity-driven fractionation strategy to identify additional components of the male sea lamprey pheromone, we adopted the procedure by Johnson et al. [15] as a bioassay and tracked the activity of an unknown component through the fractionation process. Crude extract was subjected to liquid chromatography over silica gel (150 g). Thin layer chromatograph (TLC) analysis indicated that 14 fractions were produced after elution with CHCl_3_-MeOH (10–100%). The 14 fractions were combined into four pools, excluding 3 kPZS. In the same in-stream test, the activities between synthesized 3 kPZS at 10^−12^ M and pool 3 spiked with 10^−12^ M 3 kPZS was compared. Pool 3 plus 3 kPZS retained sexually mature females for much longer than 3 kPZS alone (P<0.05) (data not shown). Fractions containing the desired compounds were combined and further purified using Sephadex LH-20 (CHCl_3_-MeOH 1∶1) to yield the compound DKPES (4.2 mg).

### Structural Analysis of DKPES

1D and 2D NMR spectra of DKPES were recorded on a Bruker Avance 900 MHz Spectrometer or a Varian 600 MHz spectrometer. Mass spectra were performed on a Q-TOF Ultima Global GAA076 LC mass spectrometer (Waters Corporation, Milford, Massachusetts, USA). Si gel (70–230 and 230–400 mesh, Merck, Darmstadt, Germany), RP-18 reverse-phase Si gel (Merck), and Sephadex LH-20 (Merck) were used for open column chromatography. TLC was conducted on glass plates precoated with GF_254_ Si gel (Merck). Spots were visualized under UV light at 254 nm and stained by spraying plates with 5% anisaldehyde acid alcoholic solution (Sigma-Aldrich, St. Louis, Missouri, USA).

### Electo-olfactogram (EOG) Recording

The olfactory potency of DKPES to female sea lamprey was determined using EOG recording. Females were exposed to solutions of L-arginine (Sigma-Aldrich), which was used as the standard odor [10], [29], 3 KPZS (Bridge Organics Co., Vicksburg, Michigan, USA; >97% purity), which was used as a positive control, and DKPES (∼90% purity). Stock solutions of 3 KPZS and DKPES were prepared using deionized water and stored at –80°C. A 10^–2^ M L-arginine stock solution was prepared using deionized water and stored at 4°C. The EOG recordings were performed as previously described [10], [30]. Briefly, females were anesthetized, immobilized, and placed in a water-filled trough with the head above water and the gills supplied with oxygenated well water. The olfactory lamellae were surgically exposed and perfused with well water. The differential electrical potential between the skin surface and lamellae in response to each stimulus was recorded by a Power Lab (ADI Instruments, Castle Hill, Australia).

Stock solutions of 3 KPZS and DKPES were diluted in well water immediately before EOG testing. To determine the concentration-response relationships, a 10^–5^ M _L_-arginine standard was introduced into the olfactory epithelium of a female sea lamprey for 5 s, and the EOG response was measured to establish a reference of electrical activity [10]. Blank well water was introduced next to determine the blank water control response. Increasing concentrations of 3 KPZS and DKPES starting at 10^–12 ^M were then introduced and the responses measured. Each trial was concluded by measuring the response to blank well water and _L_-arginine at the end of the dilution series. The olfactory epithelium of each sea lamprey was allowed to recover for 3 min between each stimulus and each concentration of 3 KPZS and DKPES was tested at least twice (a third time if olfactory sensitivity was not restored during the recovery period). The EOG response magnitudes (mV) were expressed as a percentage of the response to the L-arginine standard. Concentration response curves for 3 KPZS and DKPES were visually compared and the response threshold was determined by identifying the lowest concentration in which the response was larger than the blank water control (Student’s T test).

### In-stream Behavioral Experiments

Only sexually mature female sea lamprey were used during field trials. A 23 mm-long half duplex passive integrated transponder (PIT) tag (Oregon RFID, Portland, Oregon, USA) was fitted into a latex sleeve, and surgically attached to the mid-dorsal region of each sexually mature female using a suture on both sides (Size 3-0, Ethicon Inc., Cornelia, Georgia, USA). No anesthesia was used in this process because common fish anesthetics may disrupt olfactory-mediated behaviors [31]. Unique color combinations of ribbon tags (Hallprint Pty Ltd, Hindmarsh Valley, Australia) were attached to the anterior and posterior dorsal fin for visual observation in the field. Visual observation was used to confirm PIT observation in the field [15]. Tagged animals were immediately transferred into aerated holding tanks with a constant flow of Lake Huron water for up to 24 hours, until they were stocked into stream acclimation cages at the field site. Before tagged females were moved to the field site, they were monitored for signs of distress or mortality.

Test odorants 3 KPZS was custom synthesized by Bridge Organics (Vicksburg, Michigan, USA; purity >97%) in 2007, and stored at −80°C. A 10 mg×mL^–1^ stock solution of synthesized 3 KPZS (in 100% methanol) was prepared, vortexed, and transferred into five vials of 10-mL aliquots, each. 3 KPZS stock solution was stored at –80°C until use. The concentration of DKPES was determined by UHPLC/MS/MS with multiple reaction monitoring. UHPLC–MS/MS analyses were carried out in a Waters Acquity ultra-performance liquid chromatography system (Waters Corporation, Milford, Massachusetts, USA) with a Micromass Quattro Premier XE tandem quadruple mass spectrometer (Waters, Manchester, United Kingdom) equipped with ESI source. The analytes were separated on a C18 Acquity column (2.1 mm × 100 mm, 1.7 m) with column temperature at 50°C. The injection volume was 10 µL. The mobile phase for elution was a gradient established between solvent A (10 mM TEA in water) and solvent B (methanol) at a flow rate of 150 µL/min. Samples were kept in the autosampler at 4°C throughout the analysis. The ESI source operated in negative ion mode, and its main working parameters were set as follows: capillary voltage, 3.50 kV; extractor voltage, 5 V; source temperature, 130°C; desolvation temperature, 350°C; desolvation gas flow, 600 L/h (N_2_, 99.9% purity). Argon (99.9999% purity) was introduced as the collision gas into the collision cell at a flow rate of 0.20 mL/min. Multiple reaction monitoring (MRM) measurements transition (*m/z* 449.32>96.67) were performed using individually optimized cone voltage (58 V) and collision energy (40 eV). The dwell time established for each transition was 0.2 s, and interscan delay was set at 20 ms. Data acquisition was carried out by Masslynx 4.1 software (Waters). To mimic the composition of sea lamprey SMW, the concentration of 3 KPZS was determined following methods developed by Li et al. [32].

**Figure 4:**
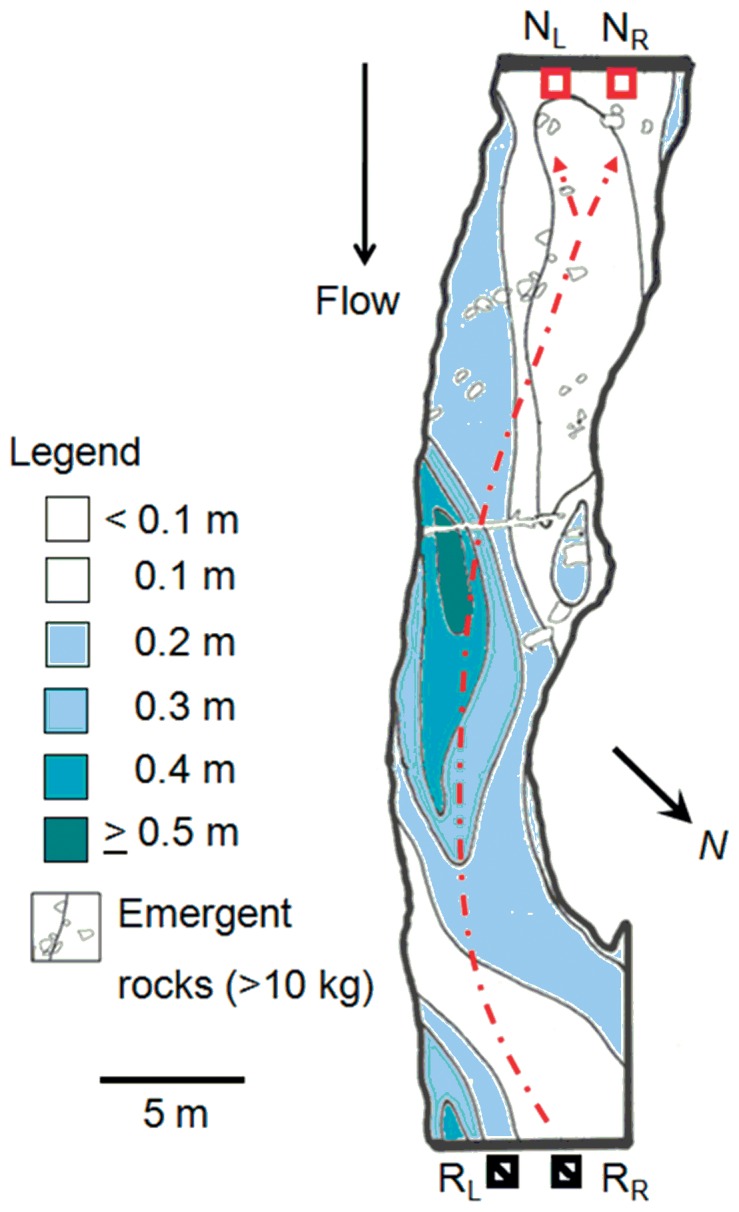
The section of the Upper Ocqueoc River, Millersburg, Michigan, USA (T35N, R3E, Sec. 27) used as the in-stream experimental site for observing nest selection of sexually mature female sea lamprey in relation to mixtures of DKPES and 3 KPZS, 3 KPZS alone, DKPES alone, and SMW. R_R_ and R_L_ indicate location of release cages downstream, and N_R_ and N_L_ are the locations of Passive Integrated Transponder (PIT) antennas where odorants were applied, and the frequency of PIT tagged female lamprey entering each nest was recorded. Depth details of the site (meters), and location of obstructive boulders (>10 kg) are shown.

Stream behavioral tests were conducted from 17 June to 27 July, 2010, and from 20 June to 29 July, 2011, to determine if DKPES is behaviorally active in natural environments. A 45 m-long stretch of riffle in the upper Ocqueoc River, Millersburg, Michigan, USA, was used to test sexually mature female sea lamprey responses to mixtures of DKPES and 3 KPZS. This area historically supported natural spawning populations of sea lamprey before a barrier was constructed at the mouth of the river that prevented their migration upstream [33]. At the upstream end of the field site, two 1 m^2^ nests, constructed of polyvinyl chloride tubing, were placed side-by-side to one another on the stream bed, 1.25 m apart. Copper wire was wrapped around each nest frame twice to make PIT antennas. Antenna scan frequencies were set to roughly 3 scans×sec^–1^. In the center of the stream channel, two release cages (0.25 m^3^) were located 45 m downstream from the side-by-side antennas (Figure 4).

Test odorants were mixed in two separate mixing bins on shore, and pumped into the center of each nest using peristaltic pumps (Masterflex 7553–70, Cole-Parmer, Vernon Hills, Illinois, USA). Odorants were administered into the center of each nest at constant rates of 167±5 mL×min^−1^ over a 2 hour period for each trial. Two trials were conducted per day between 0700 h and 1300 h.

In 2010, our specific goal was to examine whether DKPES would increase the robustness of the preference response of sexually mature females to synthesized 3 KPZS. Synthesized 3 KPZS was applied to one nest, while a mixture of synthesized 3 KPZS and DKPES was applied to the adjacent nest. The nest that received the mixture was alternated per trial. Test odorants were mixtures of DKPES and 3 KPZS at ratios of 1∶29.8, 2∶29.8, and 10∶29.8 respectively. Synthesized 3 KPZS was always applied in equal concentrations (5×10^–13^ M) to both nests per trial. Controls were conducted to insure that there was no bias between the two nests. For control trials, 3 KPZS (5×10^–13^ M) was administered to both nests. To assure the correct molar concentration of 3 KPZS was applied to the stream, stream discharge was taken every three days, or after a precipitation event, using a Marsh-McBirney portable flow meter (Flo-Mate 2000, Fredrick, Maryland, USA). The starting ratio of 1∶29.8 (DKPES:3 KPZS) was chosen based on the natural ratio that was observed in wash-water collected from sexually mature males prior to field studies. Since we have a narrow window of opportunity to test naturally mature sea lamprey in streams, and a limited volume of isolated and purified DKPES, only two other ratios of DKPES:3 KPZS could be tested (*e.g.* increasing DKPES two-fold and 10-fold). The volume of DKPES stock solution (1 mg×mL^−1^) applied to the stream for each ratio was calculated based on the volume of 3 KPZS stock solution (1 mg×mL^–1^) required to activate the stream to 5×10^–13^ M.

In 2011, we determined whether DKPES alone was sufficient to attract sexually mature females to a nest. DKPES was applied to one nest, while 100% methanol was applied to the adjacent nest. The nest that received DKPES was alternated per trial. Concentration of DKPES was kept at the same level when it is applied together with 3 kPZS at the 10∶29.8 ratio. Synthesized 3 KPZS combined with DKPES was tested against SMW to determine the preference response of sexually mature females when all odorants were applied simultaneously. A mixture of synthesized 3 KPZS and DKPES (10∶29.8) was applied to one nest while SMW was applied to the adjacent nest. A volume of SMW was applied so that the concentration of 3 KPZS was the same between both nests (5×10^−13^ M). Stream discharge measurements followed the same protocol as in 2010.

Each trial lasted for 2 hours. During the first 0.5 hour of each trial, odorants were administered to the stream while 10 sexually mature females acclimated downstream. Females were released at 0.5 hour. The proportion of test animals that entered each nest was recorded using PIT antenna nests and visual observations. Trials testing1∶29.8 treatments were conducted first, 2∶29.8 treatments second, through 10∶29.8 treatments towards the end of the field season to insure that there was enough isolated DKPES stock solution for adequate replication of each treatment. Since the natural spawning season of sea lamprey occurs over a very narrow time frame of roughly 3 weeks in the summer [33], and random flooding events are common in the Ocqueoc River, we have found this pattern of treatment testing to be the only appropriate design in our case.

### Data Analysis for In-stream Behavioral Experiments

Two main binary response variables were examined for behavioral experiments; 1) the distribution of released animals that swam upstream and entered a nest during our three treatments (0 = no enter, 1 = enter), and 2) of those animals that entered a nest, the distribution that entered the nest containing the test treatment (0 = enter control, 1 = enter treatment). Since 3 KPZS was administered into both nests during control trials, one nest was randomly assigned for statistical purposes, to be the “treatment” nest. The “treatment” nest was randomly chosen to be the right nest, and alternated every trial. Statistical analyses followed Johnson et al. [15]. Briefly, logistic regression with a binomial distribution was examined using R-software (R version 2.11.1, Vienna, Austria) to compare the response variables across the three treatment mixtures. No signs of nonlinearities or overdispersion were observed in the models. All behavioral statistics reported are two-tailed analyses (α = 0.05).

## Supporting Information

Figure S1 HR-ESI-MS of DKPES.
(TIF)

Figure S2 ^1^H NMR spectrum of compound DKPES.
(TIF)

Figure S3 ^13^C NMR spectrum of compound DKPES.
(TIF)

Figure S4 ^1^H-^1^H COSY spectrum of compound DKPES.
(TIF)

Figure S5 gHSQC spectrum of compound DKPES.
(TIF)

Figure S6 gHMBC spectrum of compound DKPES.
(TIF)

Figure S7 NOESY spectrum of compound DKPES.
(TIF)
