# Supplementary figures and images for: Characterization of a Novel Bile Alcohol Sulfate Released by Sexually Mature Male Sea Lamprey (Petromyzon marinus)
Source: PLoS One. 2013 Jul 9;8(7):e68157. doi: 10.1371/journal.pone.0068157 (PMC3706596; doi:10.1371/journal.pone.0068157)

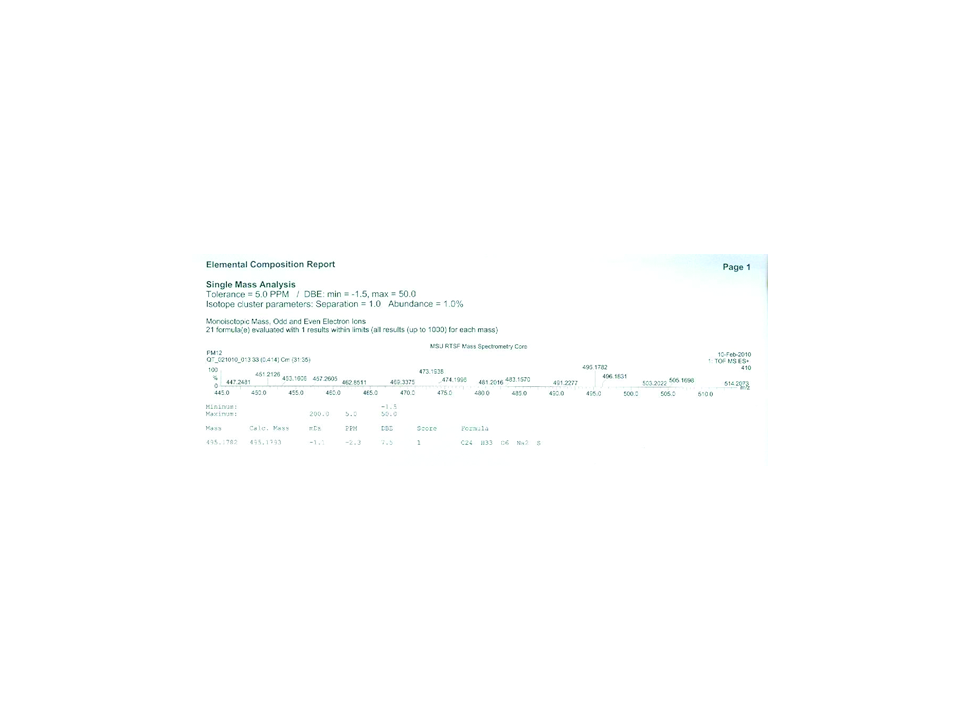

Supplement: Figure S1 — HR-ESI-MS of DKPES. (TIF) [file pone.0068157.s001.tif]

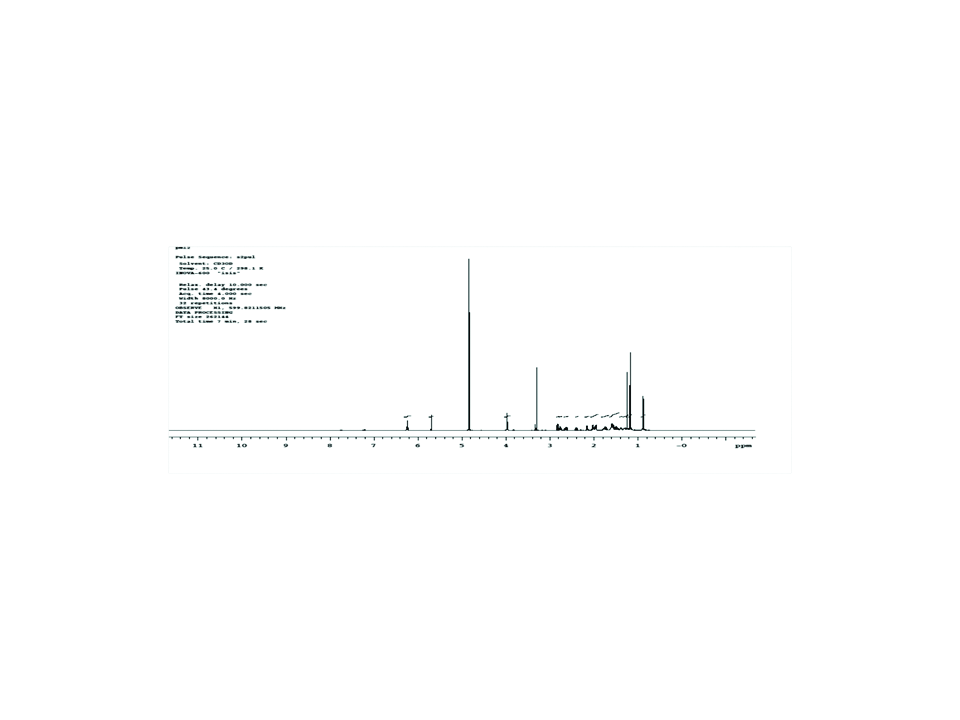

Supplement: Figure S2 — 1H NMR spectrum of compound DKPES. (TIF) [file pone.0068157.s002.tif]

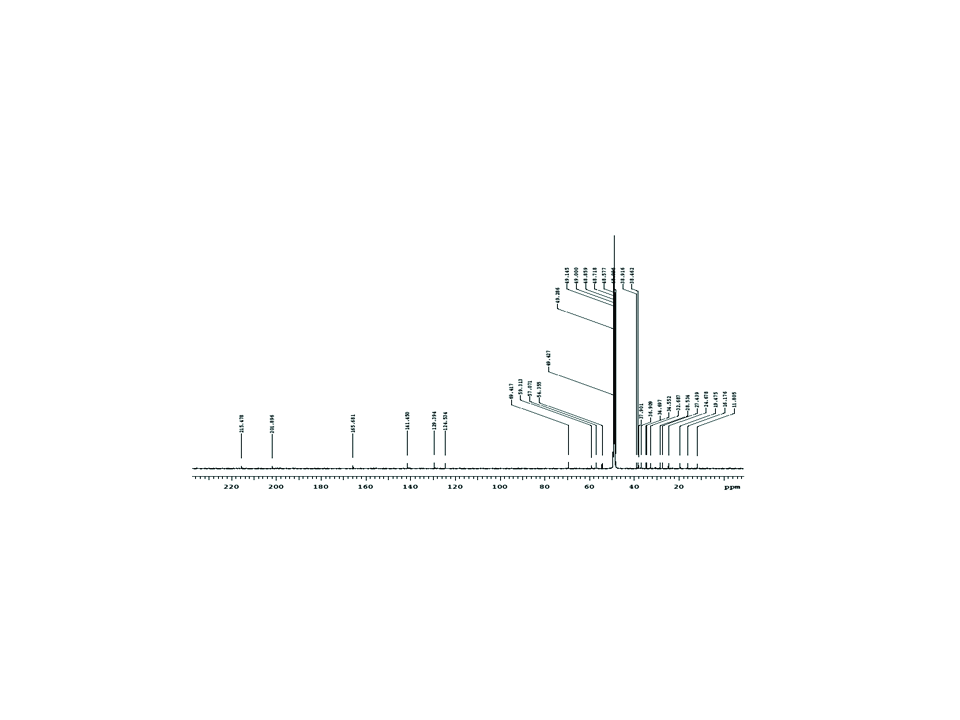

Supplement: Figure S3 — 13C NMR spectrum of compound DKPES. (TIF) [file pone.0068157.s003.tif]

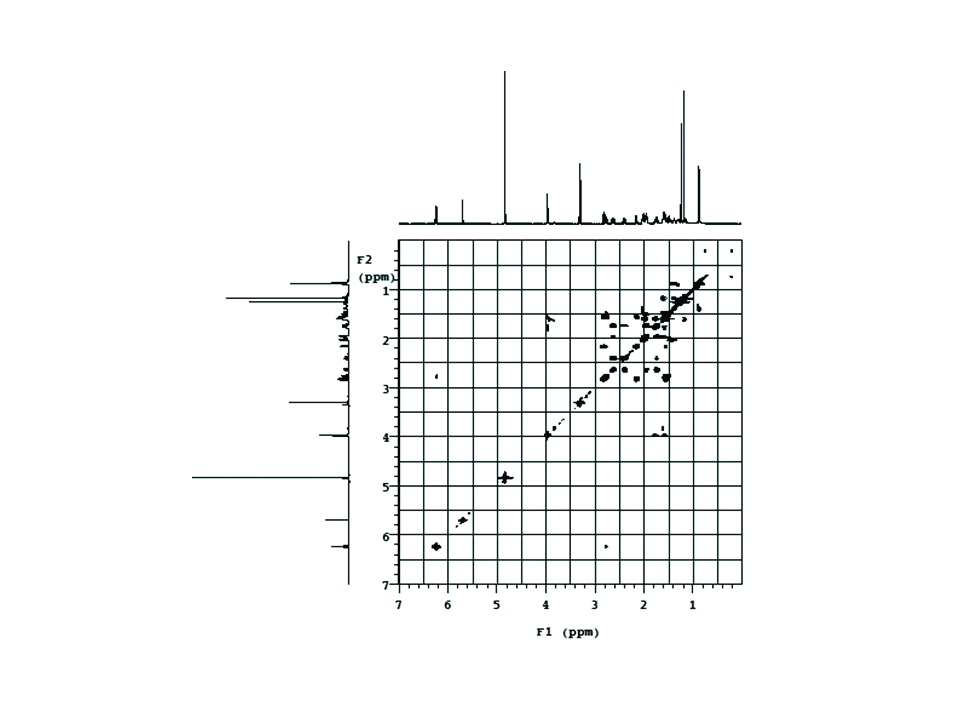

Supplement: Figure S4 — 1H-1H COSY spectrum of compound DKPES. (TIF) [file pone.0068157.s004.tif]

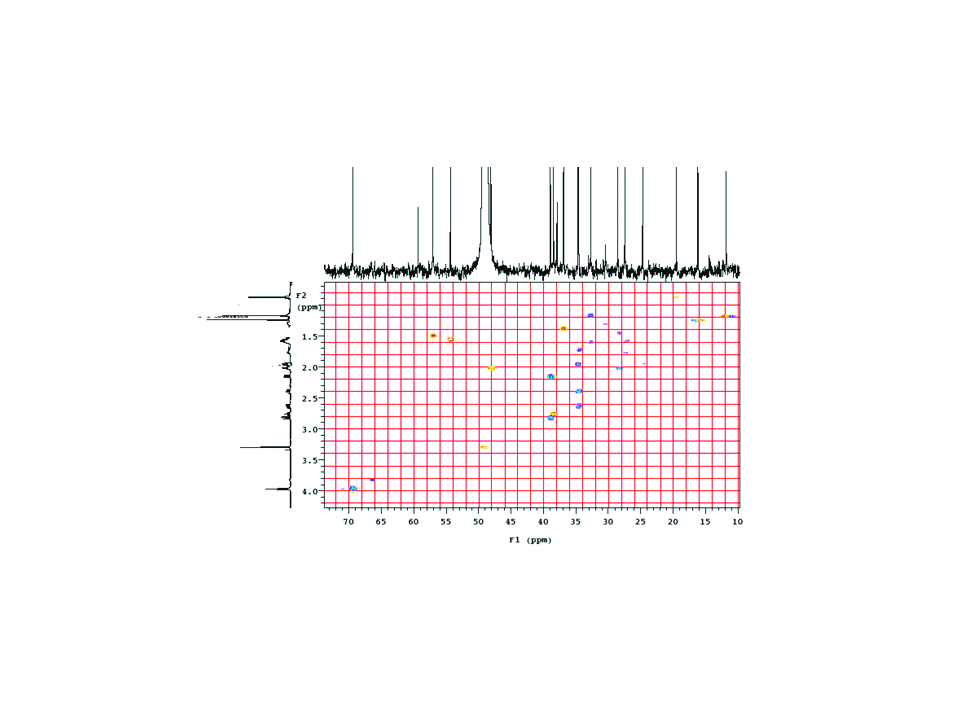

Supplement: Figure S5 — gHSQC spectrum of compound DKPES. (TIF) [file pone.0068157.s005.tif]

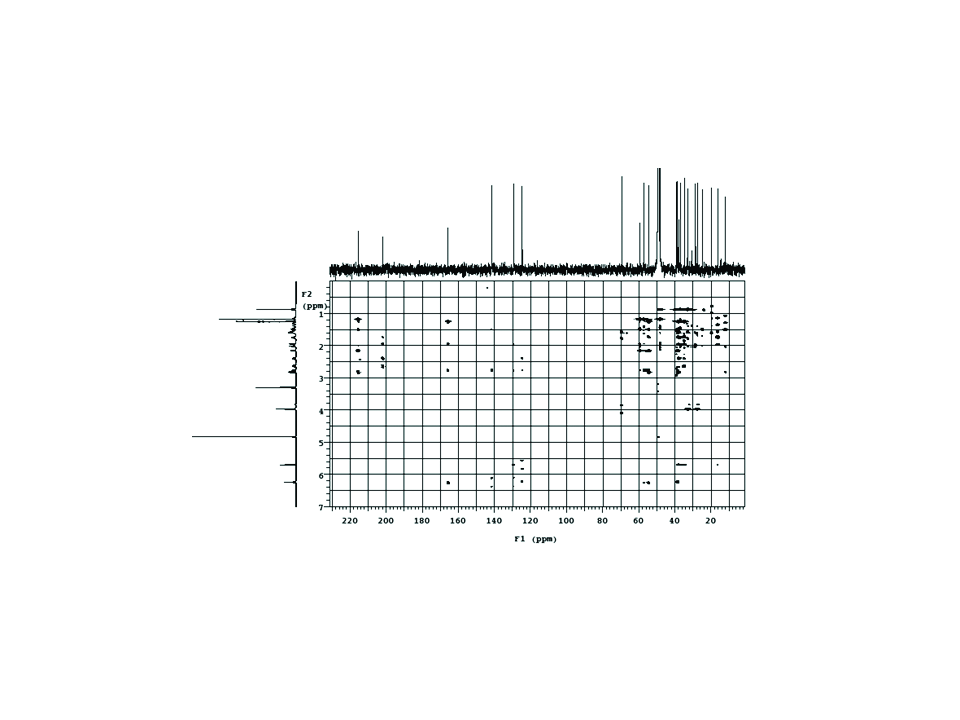

Supplement: Figure S6 — gHMBC spectrum of compound DKPES. (TIF) [file pone.0068157.s006.tif]

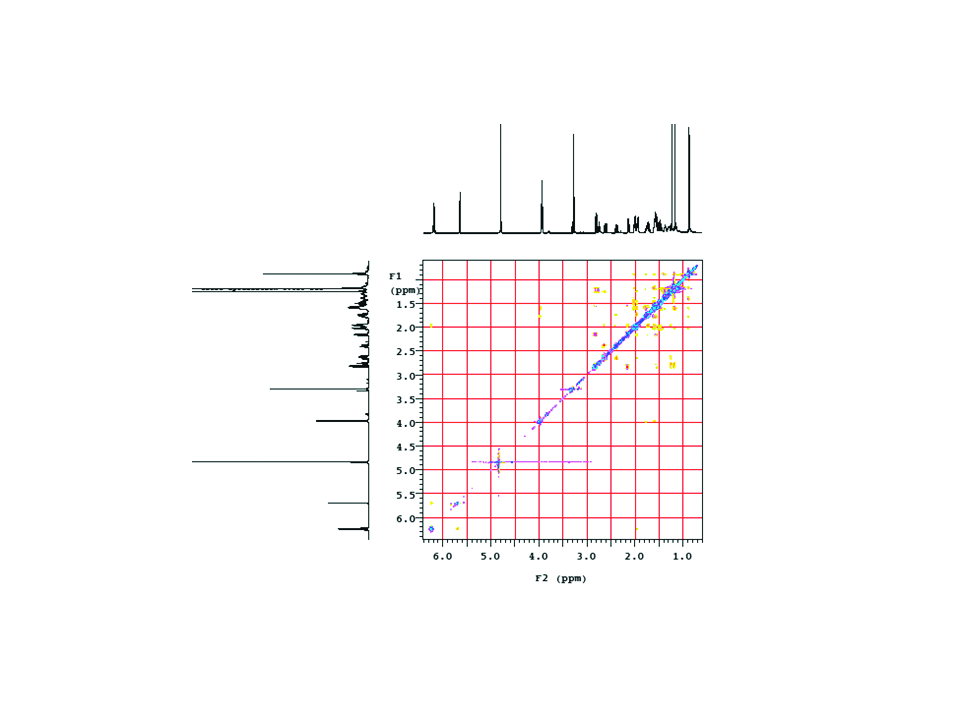

Supplement: Figure S7 — NOESY spectrum of compound DKPES. (TIF) [file pone.0068157.s007.tif]
